# A Rare Case of Primary Mediastinal Endodermal Sinus Tumor Presenting with Hemoptysis

**DOI:** 10.7759/cureus.9517

**Published:** 2020-08-02

**Authors:** Muhammad Noor, Berenice L Leal, Dhruv Patel

**Affiliations:** 1 Radiology, AdventHealth Orlando, Orlando, USA; 2 Radiology, Osceola Regional Medical Center, Kissimmee, USA

**Keywords:** yolk sac tumor, endodermal sinus tumor, non-seminomatous germ cell tumors, extragonadal germ cell tumors

## Abstract

Mediastinal non-seminomatous germ cell tumors (NSGCTs) are very rare, with an approximate annual incidence of 500 in the United States. Here, we present a case of a 22-year-old male presenting with hemoptysis who was found to have primary mediastinal NSGCT, endodermal sinus tumor type (or yolk sac type). We review the imaging findings, pathology, and treatment of primary mediastinal endodermal sinus tumors.

## Introduction

Extragonadal germ cell tumors (EGCTs) are uncommon pathologies, usually occurring in early childhood or early adulthood, with a male sex predilection [1]. They are characterized as either seminomatous or non-seminomatous types. These rare tumors have a poor prognosis, the worst of which occurs in the yolk sac and combined histopathologic tumors. Staging and thus prognosis of EGCT is based on both histopathology and imaging findings (i.e. bulky disease and distant metastatic disease) and thus, a multidisciplinary approach is best suited for the understanding and treatment of the disease [2].

## Case presentation

A 22-year-old incarcerated male with no known past medical history presented with a two-month history of hemoptysis, subjective fevers, pleuritic chest pain, and unintentional weight loss of approximately 30 pounds. He had recently emigrated from El Salvador and was incarcerated for approximately seven months prior to symptom onset.

**Figure 1:**
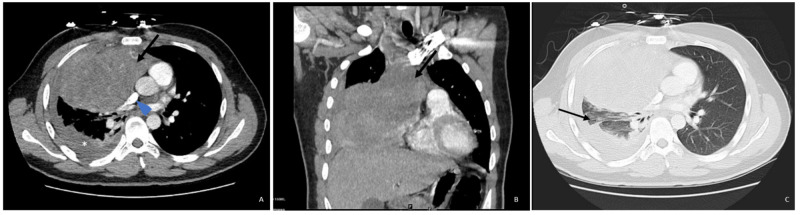
CT Chest Evaluation Axial (A) and coronal (B) contrast-enhanced CT images in soft tissue window demonstrate a large right anterior mediastinal soft tissue mass (arrows) which is largely hypodense with heterogenous enhancement. This mass is effacing the right middle lobe, narrowing of the SVC (arrowhead in A), and causing leftward displacement of the mediastinum. Axial image (A) also demonstrates a small right pleural effusion (asterisk) with adjacent atelectasis. (C) Axial contrast-enhanced CT image in lung window demonstrates the compressive atelectasis (arrow) surrounding the small right pleural effusion to a better extent. A differential based on images and patient’s age includes lymphoma, neurogenic tumor, neoplastic thymoma, or other neoplasms.

**Figure 2:**
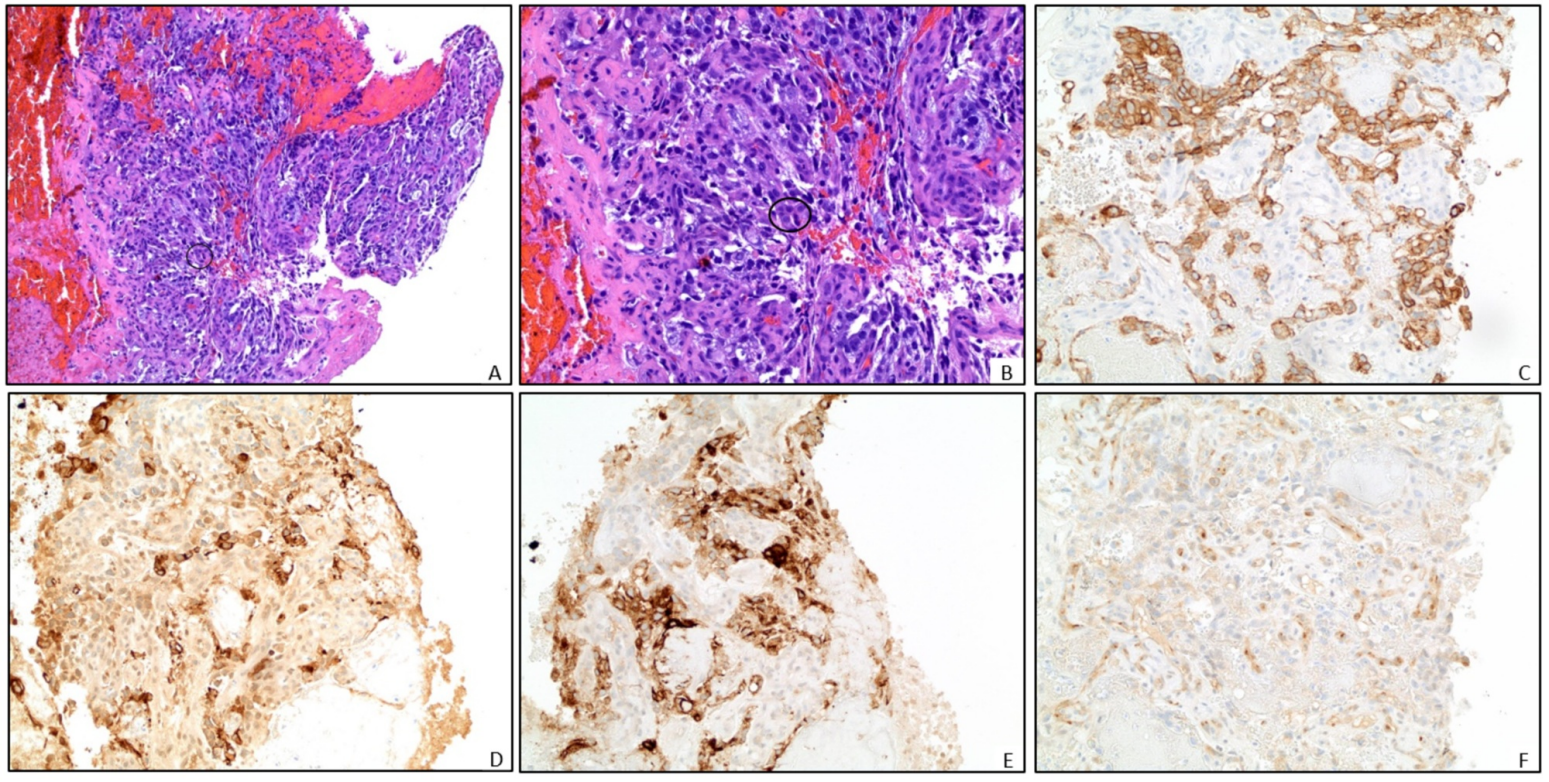
Pathology Representative pathology slides from right lung mass biopsy (A) and magnified view (B) demonstrate blood clot and necrotic tissue with rare foci of malignant cells (circled). These cells have pleomorphic nuclei, a high mitotic rate, and have an organoid to papillary architecture. There are rare cytoplasmic eosinophilic globules present. No lymphocytes are associated with the tumor. Immunohistochemical stains are as follows: (C) CK AE1/AE3 positive in tumor cells; (D) Alpha-fetoprotein (AFP) demonstrates patchy positivity; (E) GLYPICAN-3 positive; and (F) CD 117 patchy positivity. These findings, including the clinical history of an elevated AFP are consistent with a non-seminomatous germ cell tumor, yolk sac type. While no other germ cell elements are identified, the majority of this tumor is necrotic and other elements composing a mixed germ cell tumor cannot be excluded.

**Figure 3:**
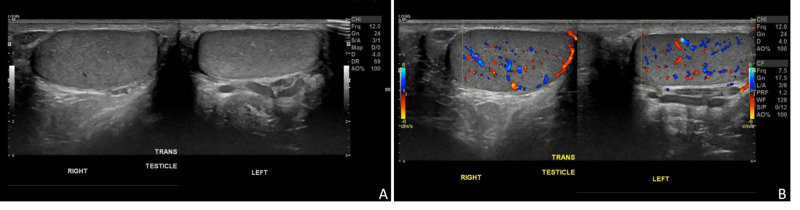
Ultrasound Evaluation Grayscale (A) and color Doppler (B) ultrasound images of the bilateral testes show no focal abnormality. This imaging examination is necessary to exclude a testicular primary in any suspicious or biopsy-proven case of extra-testicular germ cell tumors.

CT-angiography of the chest in the ED revealed a 12 cm mass in the anterior mediastinum, with mass effect on the superior vena cava (SVC), leftward displacement of the mediastinum and heart, and a right pleural effusion (Figure 1). Notable labs included normocytic anemia, leukocytosis, and an elevated alpha-fetoprotein (AFP) with a normal β-hCG. Pathology was consistent with a non-seminomatous germ cell tumor of the yolk sac type (Figure 2). A follow-up testicular ultrasound showed no focal abnormalities (Figure 3).

The patient was treated with one cycle of bleomycin, ifosfamide, cisplatin (BIP) and filgrastim chemotherapy before discharge. No additional outpatient information was available for internal assessment of the patient’s status.

## Discussion

Our patient was found to have a yolk sac tumor in the mediastinum. Mediastinal non-seminomatous germ cell tumors (NSGCTs) have an estimated annual incidence of approximately 500 in the United States. Mediastinal NSGCT commonly presents with nonspecific systemic signs, such as fever, weight loss, chest pain, cough, and hemoptysis [1]. Furthermore, presentation with Klinefelter syndrome and exogenous insulin production have also been reported [2].

EGCTs are rare and represent approximately 1-5% of all germ cell tumors [1,3]. Malignant EGCTs are grouped as either malignant seminomatous tumors (40%) or malignant non-seminomatous tumors (60%) [3]. ECGTs most commonly occur in the midline of the body and is thought to develop due to the failed migration of primary germ cells during embryogenesis, specifically, with 5-10% of all cases arising in the anterior mediastinum [4,5]. In the non-seminomatous category, yolk sac tumors have the worst prognosis. Yolk sac tumors (also known as endodermal sinus tumors) are most commonly seen as primary testicular tumors in infants and children under the age of three [2]. Whether gonadal or extragonadal, they release AFP tumor markers, and microscopic features are unequivocal, including reticular or microcystic proliferation and the characteristic Schiller-Duval bodies (which resemble primitive glomeruli) [2,5].

The treatment of extragonadal yolk sac tumours follows that of primary gonadal foci due to histologic similarities. Chemotherapeutic regimens usually include cisplatin, etoposide, and bleomycin. While surgical excision was previously not considered due to the assumption of metastasis, however, most recent studies suggest excisional surgery following successful treatment response to cisplatin-based chemotherapy [4,6]. Prognosis of germ cell tumors is based upon AFP levels, location, metastatic diseases, and the ability to have complete surgical resection [6]. Patients with a primary mediastinal focus are deemed to have a poor prognosis just based on location alone, giving them a five-year survival rate of 40-50% and a six-month survival rate following relapse [3,7].

## Conclusions

This case outlined the presentation of an unfortunate 22-year-old male who presented with hemoptysis and was found to have a large anterior mediastinal mass. Although imaging provided detailed information about the characteristics of the mass, the differential remained broad. Eventually, the mass was biopsy-proven non-seminomatous germ cell tumor, yolk sac type. Due to the absence of any additional focus of tumor and a negative testicular ultrasound, his diagnosis was deemed to be a primary mediastinal yolk sac tumor (endodermal sinus tumor). The patient was treated with a cisplatin-based chemotherapeutic regimen.

Upon examining the literature, we have found that patients usually present with nonspecific systemic signs of malignancy, chest pain, cough, and hemoptysis. Treatment usually consists of a cistplatin-based chemotherapeutic regimen followed by surgical intervention. Unfortunately, yolk sac tumors have the worst prognosis of all extra-gonadal germ cell tumors with a prognosis of less than 50% at five years.

## References

[1] McKenney JK, Heerema-McKenney A, Rouse RV (2007). Extragonadal germ cell tumors: a review with emphasis on pathologic features, clinical prognostic variables, and differential diagnostic considerations. Adv Anat Pathol.

[2] Kalhor N, Moran CA (2018). Primary germ cell tumors of the mediastinum: a review. Mediastinum.

[3] Akasbi Y, Najib R, Arifi S (2014). Complete histologic response to chemotherapy in a patient with a mediastinal yolk sac tumor: a case report. BMC Res Notes.

[4] Kesler KA, Stram AR, Timsina LR, Turrentine MW, Brown JW, Einhorn LH (2020). Outcomes following surgery for primary mediastinal nonseminomatous germ cell tumors in the cisplatin era. J Thorac Cardiovasc Surg.

[5] Liu B, Lin G, Liu J, Liu H, Shang X, Li J (2018). Primary mediastinal yolk sac tumor treated with platinum-based chemotherapy and extended resection: Report of seven cases. Thorac Cancer.

[6] Kesler KA, Rieger KM, Hammoud ZT (2008). A 25-year single institution experience with surgery for primary mediastinal nonseminomatous germ cell tumors. Ann Thorac Surg.

[7] Nakhla SG, Sundararajan S (2016). A rare case of primary anterior mediastinal yolk sac tumor in an elderly adult male. Case Rep Oncol Med.

